# Clonality, Virulence Genes, and Antimicrobial Resistance of Dairy Ruminants in Mastitic Milk-Associated *Staphylococcus aureus* in Sicily

**DOI:** 10.3390/antibiotics14020188

**Published:** 2025-02-12

**Authors:** Nunziatina Russo, Nunzio Alberto Fazio, Francesca Licitra, Joanna Gajewska, Alessandro Stamilla, Rosario Salonia, Wioleta Chajęcka-Wierzchowska, Cinzia L. Randazzo, Cinzia Caggia, Francesco Antoci, Giuseppe Cascone

**Affiliations:** 1 Di3A—Dipartimento di Agricoltura, Alimentazione e Ambiente, Università degli Studi di Catania, 95123 Catania, Italy; nunziatinarusso83@gmail.com (N.R.); nunzio.fazio@phd.unict.it (N.A.F.); alessandrostamilla@gmail.com (A.S.); cinzia.randazzo@unict.it (C.L.R.); 2 ProBioEtna srl, Spin Off of University of Catania, Via S. Sofia 100, 95123 Catania, Italy; 3 Servizio Igiene Alimenti di Origine Animale (SIAOA), Azienda Sanitaria Provinciale (ASP), Via S. Giovanni Bosco 6, 97100 Ragusa, Italy; francesca.licitra@asp.rg.it; 4 Department of Food Microbiology, Meat Technology and Chemistry, Faculty of Food Science, University of Warmia and Mazury in Olsztyn, Plac Cieszyński 1, 10-726 Olsztyn, Poland; joanna.gajewska@uwm.edu.pl (J.G.); wioleta.chajecka@uwm.edu.pl (W.C.-W.); 5 Istituto Zooprofilattico Sperimentale of Sicily, 90129 Palermo, Italy; saro.salonia77@gmail.com (R.S.); antocif@gmail.com (F.A.); giuseppe.cascone@izssicilia.it (G.C.)

**Keywords:** *Staphylococcus aureus*, antimicrobials, multi-drug resistance, MLST

## Abstract

**Background:** *Staphylococcus aureus* is one of the most prevalent pathogens causing mastitis in dairy animals and represents a serious issue of public health concern due to its resistance against multiple antimicrobials. **Objectives**: This study assessed 101 *S. aureus* isolates obtained from quarter milk of animals with subclinical mastitis in the Ragusa area (Sicily, Italy). **Methods**: Antibiotic resistance against nine antibiotics was evaluated using the Kirby–Bauer method, and the Minimum Inhibitory Concentration (MIC) values were measured for oxacillin (OXA) and vancomycin (VA). Additionally, the isolates were genetically characterized through multiplex PCR to identify the presence of *spa*, *mec*A, *mec*C, *pvl*, *van*A, *van*B, and *van*C genes, along with pulsed-field gel electrophoresis analysis and multi-locus sequence typing (MLST). **Results**: The highest rates of antibiotic resistance were found against gentamicin (47.5%) and erythromycin (29.7%), with 86.1% of strains exhibiting resistance to at least two antimicrobials and 33.7% showing resistance to three antimicrobial classes. Furthermore, the results indicated that the presence of antibiotic resistance genes was not correlated with phenotypic resistance, and a phylogenetic analysis revealed varying phenotypic resistance profiles even within the same PFGE cluster. Lastly, alongside a new allelic profile ST 9471, MLST analysis identified five additional STs clustered into three CCs, with CC5 originating from human ancestral strains through human-to-animal host transfers, making it the dominant group. **Conclusions**: This study provided valuable insights into regional trends, allowing for the identification of significant antibiotic-resistant patterns and offering an understanding of bacterial dynamics in these environments, underscoring the importance of routine resistance surveillance in dairy farms.

## 1. Introduction

*Staphylococcus aureus* is the most clinically important species among the *Staphylococcus* genus, responsible for various diseases in both humans and animals, for which antibiotic therapy may be ineffective [1,2]. Given its ability to be, at the same time, commensal, colonizing, latent, or disease-causing, thanks to its aptitude to switch between host species and from colonization to invasion, *S. aureus* is a challenging food-borne bacterium [3]. For dairy ruminants, it represents one of the most common causes of mastitis worldwide, in both clinical and subclinical forms [4,5]. Mastitis is an inflammatory response of the udder tissue of the mammary gland as a result of both microbial infections and the production of several virulence factors that may occur through different mechanisms [6].

Antimicrobial therapy is an important tool for controlling ruminant mastitis. Still, the widespread and uncontrolled use of antibiotics puts selective pressure on *S. aureus,* resulting in the emergence of multidrug-resistant strains, which reduces the treatment options [7]. In recent years, a growing concern has been related to methicillin-resistant *S. aureus* (MRSA) [8], which represents a challenging problem, having switched from the initial healthcare-associated environments (HA-MRSA) to the human community (CA-MRSA) and, more recently, to livestock (LA-MRSA). This latest emerging group of resistant strains has attracted attention for its ability to transmit from animals to humans and to reach final consumers through dairy products [9,10]. The ability of *S. aureus* to induce infection is attributed to a plethora of virulence factors, including toxins and immune evasion factors [2,11,12,13]. Therefore, staphylococci from both livestock and human origins can share several antibiotic resistance genes, such as *mec*A and/or his homolog *mec*C (*mec*A_LGA251_), responsible for the beta-lactam resistance [12,13]. Indeed, the *Staphylococcus* genus exhibits significant diversity in genetic makeup, pathogenicity, and disease-causing potential, also influenced by local environmental factors. Therefore, high variability in virulence and host adaptation, even among strains within the same species, is observed [14]. Hence, from a One Health perspective, the monitoring and surveillance of antibiotic-resistant *S. aureus* lineages in dairy farming assumes relevant importance. Within this framework, as suggested from several clinical and epidemiological studies, the *spa* typing [15], which is based on the gene amplifying the X region of the protein A gene, and the detection of the Panton–Valentine Leukocidin (PVL) in the epidemic spread and virulence escalation of MRSA strains, represent the most widely used methods [16]. In addition, the most effective molecular techniques for the epidemiological characterization of the *S. aureus* species are the pulsed-field gel electrophoresis (PFGE)—a highly precise technique that enables the genotyping of different *S. aureus* isolates through the enzymatic digestion of chromosomal DNA by *Sma*I—and the multi-locus sequence typing (MLST), which usually utilizes the sequences of internal fragments from seven housekeeping genes [17,18,19,20].

The present study focused on a population (n = 101) of *S. aureus* isolated from a quarter of milk of animals suffering from sub-clinical mastitis in different geographical sites in the Ragusa area (Sicily, Italy) to assess the phenotypic antibiotic resistance profile, as well as the presence of some resistance (*mec*A/*mec*C, *van*A/*van*B, *van*C) and virulence (*pvl*) genes, by multiplex PCR. In addition, to better understand the epidemiology, the genetic relationship among the isolates, and the population structure, PFGE and MLST were performed.

## 2. Results

### 2.1. Antimicrobial Resistance Profile

In the present study, the 101 *S. aureus* isolates were assessed for resistance to nine antimicrobial agents, and the results are reported in Table 1. The most prevalent resistance was detected against aminoglycoside—gentamicin (48 isolates; 47.5%) and macrolide—erythromycin (30 isolates; 29.7%). Of 101 isolates, 20 (19.8%) showed resistance to both linezolid and clindamycin molecules. In addition, 13 isolates (12.9%) exhibited resistance to ciprofloxacin, 8 (7.9%) to trimethoprim/sulfamethoxazole, and 6 (5.9%) to rifampicin. Moreover, the micro-dilution method revealed that 2 (2.0%) isolates showed resistance to oxacillin, indicating them as phenotypically MRSA (oxacillin MIC ≥ 4 µg/mL). Additionally, four isolates (4.0%) showed vancomycin resistance, one with a MIC value of 32 µg/mL, and three isolates with a MIC value of 4 µg/mL, hence classified as phenotypically vancomycin-resistant *Staphylococcus aureus* (VRSA) and vancomycin-intermediate *Staphylococcus aureus* (VISA), respectively (Table 1).

Among the tested isolates, 87 (86.1%) were resistant to at least two antimicrobials. Moreover, 34 (33.7%) isolates showed intermediate or complete resistance to three or more antimicrobial classes and are therefore considered multi-drug resistant (MDR) *S. aureus* (Table A1).

### 2.2. Genetic Characterization of S. aureus Isolates

**Figure 1:**
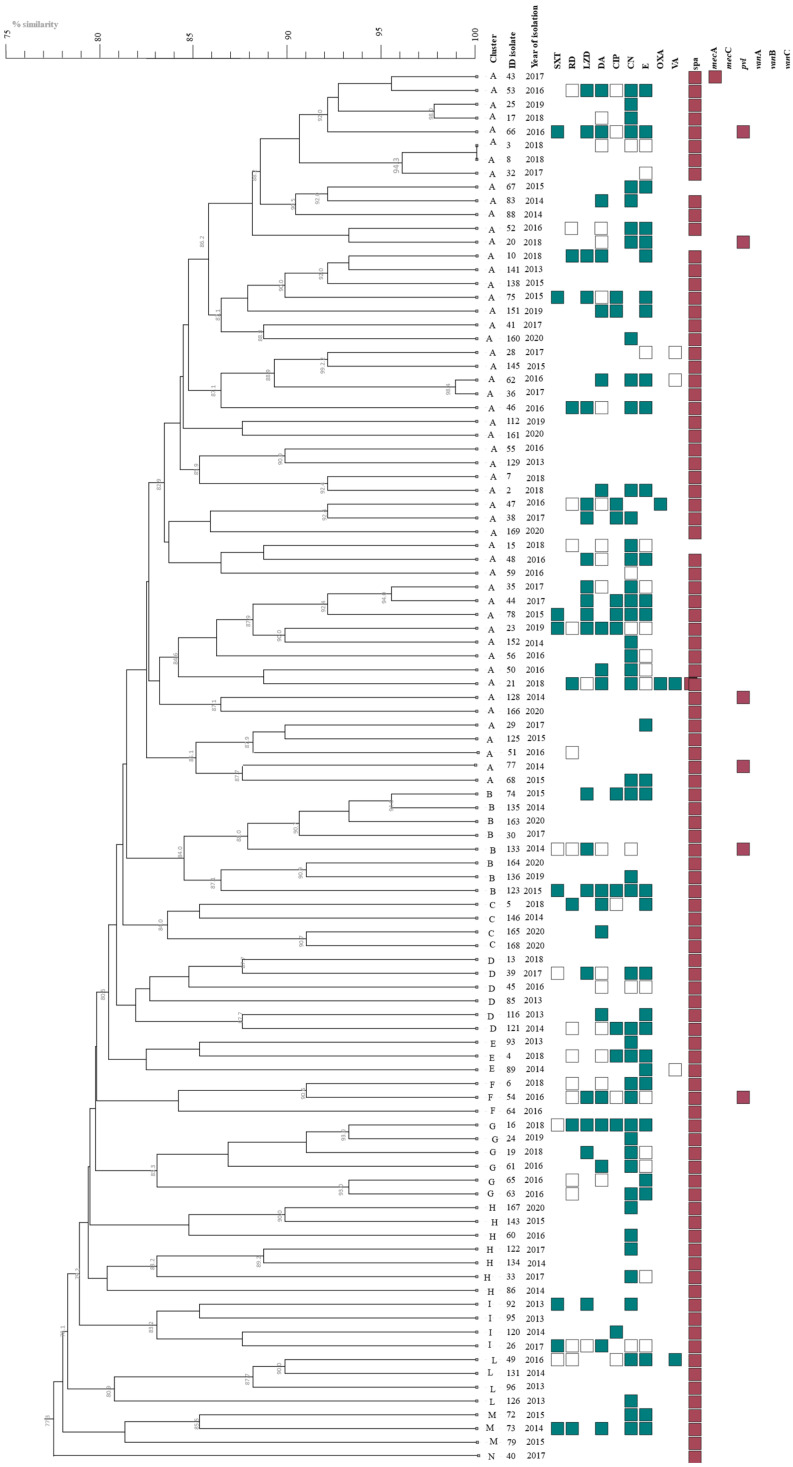
Pulsed-field gel electrophoresis (PFGE) dendrogram of *S. aureus* strains. The dendrogram was generated by Dice/UPGMA analysis (Bionumerics, Applied Maths) of *Sma*I PFGE profiles (1% optimization; 1% tolerance; cut-off point = 85%). SXT—trimethoprim/sulfamethoxazole, RD—rifampicin, LZD—linezolid, DA—clindamycin, CIP—ciprofloxacin, CN—gentamycin, E—erythromycin, OXA—oxacillin, VA—vancomycin. For antimicrobial agents: filled shape—resistance, shape outline—intermediate, shape completely omitted—sensitive; for occurrence genes: filled shape—positive, shape completely omitted—negative. The antibiogram was visualized using iTOL7.

*S. aureus* isolates were genetically characterized through a multiplex PCR colony assay, following the protocol recommended by EURL-AR, for the simultaneous detection of *spa*, *mec*A, *mec*C (*mec*ALGA251), and *pvl* genes. Out of 101 isolates, 96 (95%) were positive for *spa* typing, while the gene *pvl* was found in 5.9% of isolates (6 out of 101). Regarding the resistance genes, two isolates (namely 43 and 21, isolated from cow and goat milk samples, respectively) harbored the *mec*A gene (Figure 1). It is relevant to highlight that their antimicrobial profiles appeared quite different. In detail, the *mec*A positive strain 43 showed susceptibility for all tested antimicrobials, whereas the *mec*A positive strain 21 (Figure 1) showed resistance to five of the tested antibiotics. Instead, all the phenotypically VRSA and VISA isolates were negative for *van*A/*van*B/*van*C genes, and no isolate harbored the *mec*C gene. No significant statistical difference between strain origins and phenotypic resistance was found.

### 2.3. Clonal Relatedness and MLST Analysis

The *S. aureus* strains were typed by PFGE analysis, and the obtained profiles were subjected to BioNumerics v.7.5 (Applied Maths, Sintartens-Latem, Belgium) analysis. PFGE profiles similar at a 1.0 similarity level (=100% similarity) were considered identical. PFGE results are reported, together with the antimicrobial resistance profiles and the genetic characterization, in Figure 1. Using an 85% similarity cut-off, the strains were grouped into 12 clusters. The largest cluster, A, comprises 52 members, followed by cluster B with 8 members, and cluster H with 7 members. Clusters D and G included 6 members, whereas clusters C, I, and L included only 4 strains. Finally, with 3 members each and a single isolate, the clusters E, F, M, and N, respectively, were the least numerous clusters (Figure 1). Using the MLST analysis, successfully performed for 10 out of 12 strains, the subset of isolates was grouped in 6 different ST, comprising the new sequence type ST9471 (Table 2). Among them, ST3841 accounted for 52 isolates, followed by ST71, which grouped 25 isolates, ST352 14 isolates, ST71 11, and ST8577, which was represented by 3 isolates (Table 2). The goeBURST analysis clustered ST1, ST352, and ST9471 strains into CC97, ST1 and ST8577 into CC1, and ST3841 into CC5, making it the largest group (52 out of the 101 strains).

**Figure 2:**
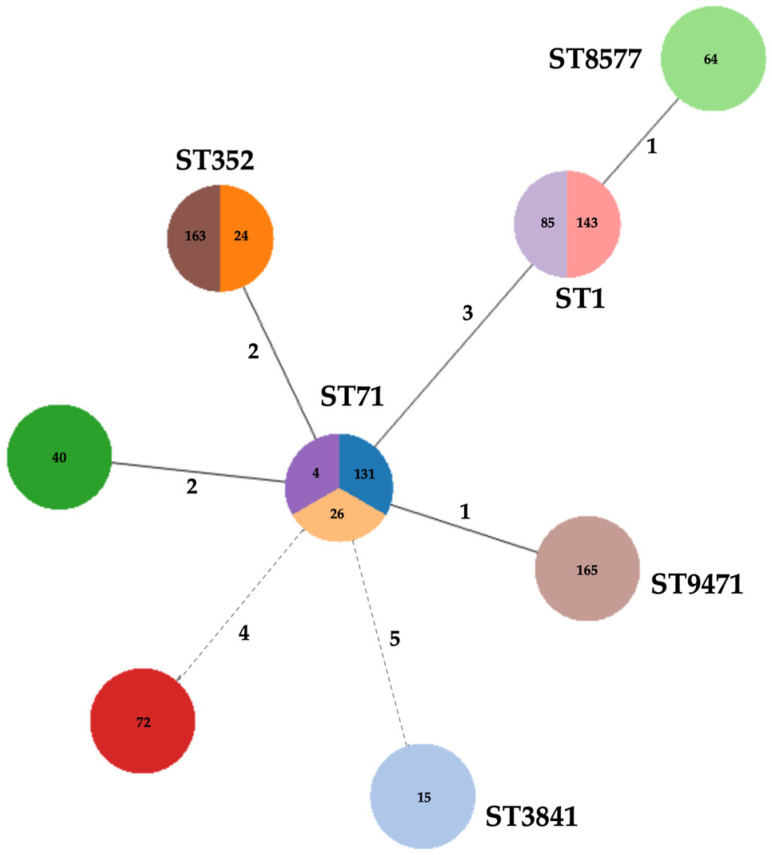
The MLST-based spanning tree of *S. aureus* isolates from cases of mastitis in the MLST database according to the ST (drawn using the Grape-Tree application). Each color corresponds to a node, representing the different STs and their septa, with the isolates sharing the same allelic profile within the same node. The values between the lines represent the number of allelic differences between two adjacent STs, and nodes with more than four allelic differences are connected by dashed lines. STs from common CCs have at least five equal alleles.

Figure 2 shows the Minimum Spanning Tree (MST)-type allele created using the Grape-Tree application, linked directly to the MLST database [21]. The following parameters were employed for the assessment of all the mastitis isolates from animals: (a) selected loci: *arc*, *aroE*, *glpF*, *gmk*, *pta*, *tpi*, and *yqil*; (b) scheme: MLST; and (c) fields: according to the desired graphic output (e.g., isolate ID, ST).

The branches in the MST figure indicate the genetic diversity and the correlation among the tested isolates of *S. aureus*.

The smaller number of divergent alleles suggested a closer genetic relationship and, thus, the presence of a dominant and common ancestral genotype, which has been efficiently transmitted over time. Indeed, the central node, with a different connection, occupied by ST71, indicates its key role in spreading traits within the analyzed population. Moreover, the grouping of several STs in a few CCs further confirmed a recent divergence from evolutionary scales.

## 3. Discussion

Notoriously recognized as a commensal, colonizing, latent organism with a high propensity to adapt to different environments [3], *S. aureus* is also an important pathogen for its high contagiousness or for inducing long-lasting chronic infections. Furthermore, due to its great ability to develop resistance against different antibiotics, it represents the most serious threat to public health [19]. Thus, the monitoring and surveillance of resistance within *S. aureus* populations in livestock environments are relevant to reduce its spread through the food chain [3].

The present study focused on a population of *S. aureus* isolated from the raw milk of several dairy animals suffering from subclinical mastitis in different locations of the Ragusa area in Sicily to determine their antimicrobial resistance profile, the presence of resistance, and virulent determinants, as well as their genetic variations. Given the diversity in genetic makeup, *S. aureus* can survive in different animal and human hosts, as well as in food, soil, and air, and increase its ability to become resistant [22,23]. As previously reported, our results showed that *S. aureus* strains exhibited high resistance to gentamycin and erythromycin, the two antimicrobials most commonly used in human and livestock production, whose frequent use and misuse can significantly contribute to resistance increase [24,25]. Erythromycin has been used for many years in the treatment of various infections as an alternative to penicillin, cephalosporin, and other beta-lactams commonly used to control staphylococcal mastitis [26], thus leading to the emergence of resistance in both animal- and farm worker-associated staphylococci [27,28]. However, the persistence of resistance to this molecule appears to be not only directly related to continued exposure to it but rather related to the exposure of commensal staphylococci with semisynthetic macrolide molecules such as clarithromycin, azithromycin, and telithromycin, clinically used to treat infections caused by bacteria other than *S. aureus,* thus contributing to erythromycin resistance commonly encountered in *S. aureus* clinical isolates [12]. Moreover, in the present study, although rare, resistance to linezolid, an oxazolidinone drug, was revealed in our *S. aureus* isolates. This resistance, also affected by the pandemic ST22-MRSA-IV clone, is rising due to the acquisition of plasmids encoding Cfr or OptrA resistance determinants, as well as mutations in the 23S rRNA genes that alter molecule-binding sites [29]. Nevertheless, linezolid remains highly active against most staphylococci, and its value in treating MRSA serious infections has been well documented [25]. *S. aureus* is known for its ability to become antibiotic-resistant, mainly through determinant acquisition via the horizontal gene transfer of mobile genetic elements and mutations. This contributes to the development and dissemination of MDR *S. aureus* in livestock, which is the most important cause of zoonotic diseases [30,31,32]. Recently, a role has also been attributed to handling and/or consuming food contaminated with MRSA, against which linezolid, and generally oxazolidinone-based drugs, are considered the agents of last resort for the treatment of nosocomial infections caused by MRSA [33,34,35]. Moreover, a small percentage of isolates showed a constitutive resistance to clindamycin, a family of macrolide–lincosamide streptogramin B (MLSB) antibiotics, which represents a reserve drug for staphylococcal infections, whose misuse has led to resistant isolates geographically distributed in the world [36]. In the present study, two MRSA strains belonging to cluster A were found positive for the *mec*A gene, and four other strains, distributed among the different clusters, were found phenotypically resistant. These results agree with previous reports that found low rates of MRSA in mastitis samples, for which the prevalence has been associated with a lack of hygienic milking measures, such as pre- and post-milking teat dip [37,38]. Despite lacking the *van* gene, several strains belonging to different clusters showed high and intermediate levels of vancomycin resistance, thus considerably phenotypically VRSA and VISA, respectively. As is well known, the glycopeptide resistance mechanisms are related to the concerted action of multiple enzymes able to modify peptidoglycan, in turn supported by gene cassettes containing a repertoire of genes and “accessory” genes that confer resistance through different mechanisms [39]. This, together with transient or reversible changes in the mechanism of action induced by various environmental and external factors [40], could explain our results.

In general, even if in the past, only sporadic reports of vancomycin-resistant MRSA have been reported; currently, the increased prevalence and incidence of VRSA in animals have been widely highlighted in Germany [41], in China [42], and in Pakistan [43,44]. Nevertheless, in agreement with other recent studies, the different resistance patterns of *S. aureus* isolates, even within the same cluster, confirm the species’ significant ability to develop resistance against one or more antimicrobial agents simultaneously through various mechanisms [43]. Furthermore, along with the substantial genetic diversity, this result, confirmed by the several obtained genotypes, including unique pulso-types, further corroborates the ability to raise distinctive geographical clusters that differ considerably among countries [29,45], as in our case, with strains coming from different Sicilian sites and different dairy animals. Although PFGE analysis revealed a heterogeneous population composed of numerous PFGE clusters and their ability to cause mastitis simultaneously in different farm animals, the MLST analysis showed low genetic variability. Indeed, it grouped the mastitis-associated *S*. *aureus* isolates into 5 STs, mainly belonging to CC1, CC97, and CC5, together including more than 89% of the tested isolates. The most numerous cluster (A) belonged to CC5, grouping the most common genotypes, not exclusively animal-related. This result could be partially explained by evolutionary proximity given by a common ancestor as the expression of effective clonal transmission, which allowed for the adaptability of pathogens to specific conditions of livestock breeding, as well as to the typical area’s environmental conditions [46,47]. Moreover, it has been widely demonstrated that dairy ruminant-associated *S. aureus* strains originated from human ancestral strains through host jumps has led to globally distributed clones such as ST1 and ST8, which have recently emerged among cattle and been identified as agents of bovine mastitis [48,49,50]. A further human-to-bovine jump of the bovine CC97 progenitor explains the CC97 prevalence as a cause of bovine mastitis worldwide, which led to a widely spread methicillin-susceptible *S. aureus* (MSSA) subpopulation and the emergence of MRSA CC97 in the community, becoming a prevalent causative agent of bovine mastitis in Europe, Asia, and the Americas, and the dominant clone in meat retail food [50,51,52]. Additionally, the newly discovered ST further confirmed the evolutionary adaptation of *S. aureus* in the Ragusa area. Finally, the low *pvl* gene presence among livestock-associated MRSA strains was confirmed here as already reported [53], although its presence was found not to be correlated to lower antibiotic resistance.

## 4. Materials and Methods

### 4.1. Milk Sample Collection and S. aureus Isolation

For this observational survey, the raw milk samples came from on-farm inspections of 1500 dairy farms in the Ragusa area, Sicily. The raw milk samples were collected at the “Milk and Mastitis Control Centre” laboratory of the Experimental Zooprophylactic Institute and analyzed. From 337 raw milk samples showing high somatic cell counts, 101 staphylococcal isolates were obtained. In detail, 90 from bovine, 6 from goats, 4 from buffalo, and 1 from sheep were collected. The isolation of *S. aureus* and its preliminary identification was performed following the institute’s internal procedures. The milk samples (0.01 mL) were plated on Brain--Heart Infusion agar (Liofilchem srl, Roseto degli Abruzzi, TE, Italy) and aerobically incubated for 24–48 h at 37 °C. Bacteria were identified based on colony morphology and Gram staining. For Gram-positive cocci, both the catalase test with hydrogen peroxide (3%) and the coagulase test, using sterile rabbit plasma, were performed. *S. aureus* was confirmed through the API Staph (Biomerieux, Craponne, France) test coupled with the specific software (VITEK 2 Systems, version 08.02), according to the manufacturer’s instructions. The *S. aureus* isolates were stored at −80 °C in cryobeads (Cryobank, Mast Group Ltd., Bootle, UK) until further analysis.

### 4.2. In Vitro Antimicrobial Susceptibility Tests

The 101 *S. aureus* isolates were tested for antibiotic susceptibility against seven antibiotics using the disk-diffusion method, following the Clinical Laboratory Standard Institute (CLSI) [54] and the EUCAST [55] guidelines. Moreover, susceptibility to oxacillin (OXA) and vancomycin (VA) for all tested isolates was performed by the micro-broth dilution method, using 96-well bottom polystyrene plates, as previously described [56]. Following the standard protocol, the isolates were tested by the Kirby–Bauer method against the seven molecules: trimethoprim/sulfamethoxazole (SXT-25 µg), rifampicin (RD-5 µg), linezolid (LZD-30µg), clindamycin (DA-2 µg), ciprofloxacin (CIP-5 µg), gentamycin (CN-10 µg), and erythromycin (E-15 µg). Single pure colonies from overnight cultures were suspended in 0.9% saline solution to obtain turbidity equal to the McFarland 0.5 and smeared on Mueller–Hinton agar plates (Liofilchem srl, Roseto degli Abruzzi, TE, Italy). Antibiotic disks were firmly applied on the inoculated plates, which were incubated for 24 h at 36 ± 1 °C. Isolates were classified into resistant (R), susceptible (S), and intermediate (I), according to the corresponding inhibition zones. *S. aureus* ATCC 25923 and 29213 were used as reference strains. All experiments were carried out in duplicate.

### 4.3. Detection of spa, mecA, mecC, pvl, and van Genes by Multiplex PCR

*S. aureus* isolates were grown overnight on BHI broth and then smeared in MSA (Mannitol Salt Agar, Liofilchem srl, Roseto degli Abruzzi, TE, Italy) plates. Once grown, individual colonies were taken and inoculated into 1.5 mL Eppendorf tubes containing 20 µL of distilled water and used as DNA templates. Genetic characterization was performed by multiplex PCR colony, following the protocol recommended by EURL-AR (EU Reference Laboratory for Antimicrobial Resistance) [57,58]. The Reaction Mix (25 µL) was prepared into a 1.5 mL microcentrifuge tube by mixing DreamTaq™ Green PCR Master Mix 2X (Thermo Fisher Scientific, Waltham, MA, USA), primers for the amplification of *spa*, *mec*A, *mec*C (*mec*ALGA251), and *pvl* genes (Table 3), ultrapure water (DNAse/RNAse free) and 2 µL of a previously extracted DNA template. The amplification program included an initial denaturation at 95 °C for 5 min followed by 30 cycles of amplification at 94 °C for 30 s, annealing at 59 °C for 1 min, and elongation at 72 °C for 1 min. Finally, an elongation step was performed at 72 °C for 10 min. The *pvl* and *mec* genes’ positive and negative *S. aureus* strains (ATCC 29213, ATCC 33591, and ATCC 49775) were used as reference strains. *Van* gene detection was performed as previously reported by Russo and coworkers [59]. Amplified products were visualized in a 2% TBE 1X agarose gel containing 3 μL of Red Nucleic Acid gel stain (Biotium, Fremont, CA, USA), and DNA molecular weight marker 100 bp (Invitrogen-Life Technologies, Carlsbad, CA, USA) was used as standard for DNA sizes.

### 4.4. Pulsed-Field Gel Electrophoresis (PFGE)

The macro-restriction fragment separation by PFGE was performed using the standardized PFGE protocol for oxacillin-resistant *S. aureus,* found on PulseNet (OPN) (Centers for Disease Control and Prevention) [60], to analyze clonal relationships among *S. aureus* strains. Plug preparation was performed as previously reported by Russo et al. [61], with an additional treatment with lysostaphin to improve the cell lysis. The digestion was carried out with 3 µL of *Sma*I restriction enzyme (Fermentas, EU) for 4 h at 25 °C, after pre-digestion in the buffer at 25 °C for 30 min. DNA fragments were resolved in 1% agarose gel in 0.5X TBE electrophoresis buffer at 14 °C using the CHEF-DR III system (Bio-Rad Laboratories, Hercules, CA, USA). The runtime was 21 h, with a constant voltage of 6 V/cm, using a linear pulse ramp of 5–40 s. PFGE band patterns were generated by BioNumerics v. 7.5 software (Applied Maths, Sint-Martens-Latem, Belgium) with a tolerance position of 1%. Clustering was based on the unweighted pair group method with arithmetic averages (UPGMA). The Dice correlation coefficient was used to detect the similarities of banding patterns. PFGE patterns similar to each other at the 1.0 similarity level (=100% similarity) were considered identical.

### 4.5. Multi-Locus Sequence Typing (MLST)

MLST was performed by amplifying the internal fragments of 7 housekeeping genes (*arc*C, *aro*E, *glp*F, *gmk*, *pta*, *tpi*, and *yqil*), according to the distribution of alleles and sequence types (STs) found in the MLST database [21]. The amplified PCR products were sequenced using the Sanger sequencing method at Microbion srl (San Giovanni Lupatoto, VR, Italy). Each isolate’s sequence type (ST) was determined by comparing the obtained sequences within the available MLST database. Strains of *S. aureus* were grouped into the same CC when they showed identical sequences in five of the seven housekeeping genes, and the dominant ST type of each CC was the ST with the highest number of variations in an individual locus [17]. MRSA isolates with unanticipated fragments or lacking fragments by multiplex PCR were defined as non-typeable (NT).

### 4.6. Statistical Analysis

All statistical analyses were performed using Prism 9.3.1. (Prism, GraphPad Software, San Diego, CA, USA), through the Kruskal–Wallis ANOVA test. For the analysis, *p* ≤ 0.05 was considered significant.

## 5. Conclusions

In the present study, the high incidence of resistance against critically important antimicrobial agents raises concern about the antibiotic resistance of *S. aureus* species. Furthermore, this study provided insight into the phylogenetic relationship among staphylococci in Sicilian dairy ruminant farms, demonstrating that raw milk might be a source of both MDR and virulent strains and that some lineages may result from human-to-animal host-switching events. The observed diversity in both pulso-types and multidrug-resistant profiles among strains, even within the same pathogenic lineage, confirms the high genomic plasticity of the species and, in turn, the high probability of horizontal gene transfer. The convergent evolution, evidenced by both the low number of CCs and the newly discovered ST, further confirms the species’ great ability to adapt in response to environmental challenges, making the dairy farm a potential reservoir of pathogenic bacteria that can rapidly switch between animals and humans and vice versa. Thus, investigations and surveillance programs are mandatory to understand the role of pathogenic transmissible clones, to recognize new patterns of virulence, and to identify the risk factors associated with antibiotic resistance spread. Although focused on a subset of strains, our study provided valuable insights into regional trends, allowing for the identification of significant patterns and offering an understanding of bacterial dynamics in these environments.

## Figures and Tables

**Table 1:** Antimicrobial resistance profiles of *Staphylococcus aureus* isolates against nine tested antimicrobial agents.

Antimicrobial Molecules	*S. aureus* Isolates (*n* = 101)					
	Resistant		Intermediate		Sensitive	
	n	%	n	%	n	%
SXT	8	7.9	4	3.96	89	88.1
RD	6	5.9	15	14.5	80	79.2
LZD	20	19.8	2	1.98	79	78.2
DA	19	18.8	18	17.8	64	63.4
CIP	13	12.9	5	4.95	83	82.2
CN	48	47.5	7	6.93	46	45.5
E	30	29.7	17	16.8	54	53.5
OXA	2	1.98	na	na	99	98.0
VA	1	1.00	3	3.00	97	96.0

SXT: trimethoprim/sulfamethoxazole; RD: rifampicin; LZD: linezolid; DA: clindamycin; CIP: ciprofloxacin; CN: gentamycin; E: erythromycin; OXA: oxacillin; VA: vancomycin. na: not applicable.

**Table 2:** MLST distribution of *S. aureus* isolates.

ST	Clonal Complex	N. of Isolates (%)	Allelic Profile						
			*arcC*	*aroE*	*glpF*	*gmk*	*pta*	*tpi*	*yqiL*
ST3841	5	52(51.5%)	12	4	1	4	12	403	3
ST352	97	14(13.9%)	3	78	1	1	1	5	3
ST1	1	13(12.9%)	1	1	1	1	1	1	1
ST71	97	11(10.9%)	18	1	1	1	1	5	3
ST9471 *	97	4(3.96%)	18	1	1	1	787	5	3
ST8577	1	3(2.97%)	386	1	1	1	1	1	1
-	-	3(2.97%)	960	1	-	37	978	5	3
-	-	1(0.99%)	18	1	1	1	12	5	-

- Not Defined. * New ST.

**Table 3:** Primer sequences and expected amplicon sizes.

Primer	Primer # (EURL-AR)	Sequence	Amplicon Size (pb)	Reference
*spa*-1113F	2819	5′-TAAAGACGATCCTTCGGTGAGC-3′	180–600	[57,58]
*spa*-1514R	2820	5′-CAGCAGTAGTGCCGTTTGCTT-3′		
*mecA* P4	2821	5′-TCCAGATTACAACTTCACCAGG-3′	162	[57,58]
*mecA* P7	2822	5′-CCACTTCATATCTTGTAACG-3′		
*pvl*-F	2823	5′-GCTGGACAAAACTTCTTGGAATAT-3′	83	[57,58]
*pvl*-R	2824	5′-GATAGGACACCAATAAATTCTGGATTG-3′		
*EA1*(+)		5′-GGGAAAACGACAATTGC-3′	732	[59]
*EA2*(−)		5′-GTACAATGCGGCCGTTA-3′		
*EB3*(+)		5′-ACGGAATGGGAAGCCGA-3′	647	[59]
*EB4*(−)		5′-TGCACCCGATTTCGTTC-3′		
*arcC*F		5′-TTGATTCACCAGCGCGTATTGTC-3′	456	[21]
*arcC*R		5′-AGGTATCTGCTTCAATCAGCG-3′		
*aroE*F		5′-ATCGGAAATCCTATTTCACAT TC-3′	456	[21]
*aroE*R		5′-GGTGTTGTATTAATAACGATATC-3′		
*glpF*F		5′-CTAGGAACTGCAATCTTAATCC-3′	465	[21]
*glpF*R		5′-TGGTAAAATCGCATGTCCAATTC-3′		
*gmk*F		5′-ATCGTTTTATCGGGACCATC-3′	417	[21]
*gmkR*		5′-TCATTAACTACAACGTAATCGTA-3′		
*pta*F		5′-GTTAAAATCGTATTACCTGAAGG-3′	474	[21]
*pta*R		5′-GACCCTTTTGTTGAAAAGCTTAA-3′		
*tpi*F		5′-TCGTTCATTCTGAACGTCGTGAA-3′	402	[21]
*tpi*R		5′-TTTGCACCTTCTAACAATTGTAC-3′		
*yqil*F		5′-CAGCATACAGGACACCTATTGGC-3′	516	[21]
*yqil*R		5′-CGTTGAGGAATCGATACTGGAAC-3′		

## Data Availability

The original contributions presented in this study are included in the article. Further inquiries can be directed at the corresponding author.

## Appendix A

**Table A1:** Detailed antimicrobial resistance and virulent profiles of *Staphylococcus aureus* isolates.

				DISC DIFUSSION METHOD							MIC					
Strain	Year	Animal	Isolation Matrix	SXT	RD	LZD	DA	CIP	CN	E	OXA	VA	spa	*mec*A	*mec*C	pvl
2	2018	Bovine	Milk	S	S	S	R	S	R	R	0.25	2	+	−	−	−
3	2018	Bovine	Milk	S	S	S	I	S	I	I	0.25	2	+	−	−	−
4	2018	Bovine	Milk	S	I	S	I	R	R	R	0.25	1	+	−	−	−
5	2018	Goat	Milk	S	R	S	R	I	S	R	0.25	1	+	−	−	−
6	2018	Bovine	Milk	S	I	S	I	S	R	R	0.25	2	+	−	−	−
7	2018	Bovine	Milk	S	S	S	S	S	S	S	0.5	2	+	−	−	−
8	2018	Bovine	Milk	S	S	S	S	S	S	S	0.5	2	+	−	−	−
10	2018	Bovine	Milk	S	R	R	R	S	S	R	0.063	1	+	−	−	−
13	2018	Bovine	Milk	S	S	S	S	S	S	S	0.5	2	+	−	−	−
15	2018	Bovine	Milk	S	I	S	I	S	R	I	0.125	0.5	−	−	−	−
16	2018	Bovine	Milk	I	R	R	R	R	R	R	0.125	1	+	−	−	−
17	2018	Bovine	Milk	S	S	S	I	S	R	S	0.25	2	+	−	−	−
19	2018	Bovine	Milk	S	S	R	S	S	R	I	0.5	2	+	−	−	−
20	2018	Bovine	Milk	S	S	S	I	S	R	R	0.5	2	−	−	−	+
21	2018	Goat	Milk	S	R	I	R	S	R	I	4	32	−	+	−	−
23	2019	Bovine	Milk	R	I	R	R	R	I	I	0.125	1	+	−	−	−
24	2019	Bovine	Milk	S	S	S	S	S	R	S	0.063	1	+	−	−	−
25	2019	Bovine	Milk	S	S	S	S	S	R	S	0.25	2	+	−	−	−
26	2017	Bovine	Milk	R	I	I	R	S	I	I	0.25	1	+	−	−	−
28	2017	Bovine	Milk	S	S	S	S	S	S	I	0.25	4	+	−	−	−
29	2017	Bovine	Milk	S	S	S	S	S	S	R	0.25	1	+	−	−	−
30	2017	Bovine	Milk	S	S	S	S	S	S	S	0.5	2	+	−	−	−
32	2017	Bovine	Milk	S	S	S	S	S	S	I	0.25	1	+	−	−	−
33	2017	Goat	Milk	S	S	S	S	S	R	I	0.063	2	+	−	−	−
35	2017	Bovine	Milk	S	S	R	I	S	R	I	0.5	2	+	−	−	−
36	2017	Bovine	Milk	S	S	S	S	S	S	S	0.063	1	+	−	−	−
38	2017	Sheep	Milk	S	S	R	S	R	R	S	0.063	1	+	−	−	−
39	2017	Bovine	Milk	I	S	R	I	S	R	R	0.063	0.5	+	−	−	−
40	2017	Bovine	Milk	S	S	S	S	S	S	S	0.063	2	+	−	−	−
41	2017	Goat	Milk	S	S	S	S	S	S	S	0.25	2	+	−	−	−
43	2017	Bovine	Milk	S	S	S	I	R	I	I	0.5	2	−	+	−	−
44	2017	Bovine	Milk	S	S	R	S	R	R	R	0.063	1	+	−	−	−
45	2016	Bovine	Milk	S	S	S	I	S	I	I	0.063	2	+	−	−	−
46	2016	Bovine	Milk	S	R	R	I	S	R	R	0.063	0.25	+	−	−	−
47	2016	Goat	Milk	S	I	R	I	S	S	R	0.125	0.5	+	−	−	−
48	2016	Bovine	Milk	S	S	R	I	S	R	R	0.25	2	+	−	−	−
49	2016	Bovine	Milk	I	I	S	S	I	R	R	0.5	4	+	−	−	−
50	2016	Bovine	Milk	S	S	S	R	S	R	I	0.25	2	+	−	−	−
51	2016	Bovine	Milk	S	I	S	S	S	S	S	0.25	0.25	+	−	−	−
52	2016	Bovine	Milk	S	I	S	I	S	R	R	0.25	2	+	−	−	−
53	2016	Bovine	Milk	S	I	R	R	I	R	I	0.063	2	+	−	−	−
54	2016	Bovine	Milk	S	I	R	R	I	R	I	0.5	2	+	−	−	+
55	2016	Goat	Milk	S	S	S	S	S	S	S	2	2	+	−	−	−
56	2016	Bovine	Milk	S	S	S	S	S	R	I	0.063	1	+	−	−	−
59	2016	Bovine	Milk	S	S	S	S	S	I	S	0.063	2	+	−	−	−
60	2016	Bovine	Milk	S	S	S	S	S	R	S	0.25	2	+	−	−	−
61	2016	Bovine	Milk	S	S	S	R	S	R	I	0.25	2	+	−	−	−
62	2016	Bovine	Milk	S	S	S	R	S	R	R	0.25	4	+	−	−	−
63	2016	Bovine	Milk	S	I	S	S	S	R	R	0.125	2	+	−	−	−
64	2016	Bovine	Milk	S	S	S	S	S	R	S	0.063	0.25	+	−	−	−
65	2016	Bovine	Milk	S	I	S	I	S	S	R	0.25	2	+	−	−	−
66	2016	Bovine	Milk	R	S	R	R	I	R	R	0.25	2	+	−	−	+
67	2015	Bovine	Milk	S	S	S	S	S	R	R	0.25	2	−	−	−	−
68	2015	Bovine	Milk	S	S	S	S	S	R	R	0.25	2	+	−	−	−
72	2015	Bovine	Milk	S	S	S	S	S	R	R	0.063	0.5	+	−	−	−
73	2014	Buffalo	Milk	R	R	S	R	S	R	R	0.25	2	+	−	−	−
74	2015	Bovine	Milk	S	S	R	S	R	R	R	0.25	2	+	−	−	−
75	2015	Bovine	Milk	R	S	R	I	R	S	R	0.063	1	+	−	−	−
77	2014	Bovine	Milk	S	S	S	S	S	S	S	0.063	1	+	−	−	+
78	2015	Bovine	Milk	R	S	R	S	R	R	R	0.25	1	+	−	−	−
79	2015	Bovine	Milk	S	S	S	S	S	S	S	0.5	1	+	−	−	−
83	2014	Buffalo	Milk	S	S	S	R	S	R	S	0.125	2	+	−	−	−
85	2013	Bovine	Milk	S	S	S	S	S	S	S	0.063	1	+	−	−	−
86	2014	Bovine	Milk	S	S	S	S	S	S	S	0.25	1	+	−	−	−
88	2014	Buffalo	Milk	S	S	S	S	S	S	S	0.5	1	+	−	−	−
89	2014	Buffalo	Milk	S	S	S	S	S	R	S	4	2	+	−	−	−
92	2013	Bovine	Milk	R	S	R	S	S	R	S	0.125	1	+	−	−	−
93	2013	Bovine	Milk	S	S	S	S	S	R	S	0.063	1	+	−	−	−
95	2013	Bovine	Milk	S	S	S	S	S	S	S	0.5	2	+	−	−	−
96	2013	Bovine	Milk	S	S	S	S	S	S	S	0.5	2	+	−	−	−
112	2019	Bovine	Milk	S	S	S	S	S	S	S	0.063	2	+	−	−	−
116	2013	Bovine	Milk	S	S	S	R	S	S	R	0.063	0.25	+	−	−	−
120	2014	Bovine	Milk	S	S	S	S	R	S	S	0.063	2	+	−	−	−
121	2014	Bovine	Milk	S	I	S	I	R	R	R	0.5	1	+	−	−	−
122	2017	Bovine	Milk	S	S	S	S	S	R	S	0.063	1	+	−	−	−
123	2015	Bovine	Milk	R	S	R	R	R	R	R	0.25	1	+	−	−	−
125	2015	Bovine	Milk	S	S	S	S	S	S	S	0.063	2	+	−	−	−
126	2013	Bovine	Milk	S	S	S	S	S	R	S	0.063	2	+	−	−	−
128	2014	Bovine	Milk	S	S	S	S	S	S	S	2	2	+	−	−	+
129	2013	Bovine	Milk	S	S	S	S	S	S	S	0.25	2	+	−	−	−
131	2014	Bovine	Milk	S	S	S	S	S	S	S	0.063	2	+	−	−	−
133	2014	Bovine	Milk	I	I	R	I	S	I	S	0.125	2	+	−	−	+
134	2014	Bovine	Milk	S	S	S	S	S	S	S	0.063	1	+	−	−	−
135	2014	Bovine	Milk	S	S	S	S	S	S	S	0.063	2	+	−	−	−
136	2019	Bovine	Milk	S	S	S	S	S	R	S	0.25	2	+	−	−	−
138	2015	Bovine	Milk	S	S	S	S	S	S	S	0.063	1	+	−	−	−
141	2013	Bovine	Milk	S	S	S	S	S	S	S	0.063	2	+	−	−	−
143	2015	Bovine	Milk	S	S	S	S	S	S	S	0.25	1	+	−	−	−
145	2015	Bovine	Milk	S	S	S	S	S	S	S	0.063	2	+	−	−	−
146	2014	Bovine	Milk	S	S	S	S	S	S	S	1	2	+	−	−	−
151	2019	Bovine	Milk	S	S	S	R	R	S	R	0.5	2	+	−	−	−
152	2014	Bovine	Milk	S	S	S	S	S	R	S	1	2	+	−	−	−
160	2020	Bovine	Milk	S	S	S	S	S	R	S	0.5	2	+	−	−	−
161	2020	Bovine	Milk	S	S	S	S	S	S	S	1	2	+	−	−	−
163	2020	Bovine	Milk	S	S	S	S	S	S	S	0.063	1	+	−	−	−
164	2020	Bovine	Milk	S	S	S	S	S	S	S	0.25	2	+	−	−	−
165	2020	Bovine	Milk	S	S	S	R	S	S	S	0.063	2	+	−	−	−
166	2020	Bovine	Milk	S	S	S	S	S	S	S	0.063	1	+	−	−	−
167	2020	Bovine	Milk	S	S	S	S	S	R	S	0.25	2	+	−	−	−
168	2020	Bovine	Milk	S	S	S	S	S	S	S	0.5	2	+	−	−	−
169	2020	Bovine	Milk	S	S	S	S	S	S	S	0.063	2	+	−	−	−
